# Delayed ovulation in cattle: Physiological mechanisms, diagnostic approaches, and reproductive management strategies

**DOI:** 10.14202/vetworld.2026.3081-3108

**Published:** 2026-07-20

**Authors:** Langgeng Priyanto, Imam Mustofa, Aswin Rafif Khairullah, Rimayanti Rimayanti, Deddy Fachruddin Kurniawan, Agung Budiyanto, Oktora Dwi Putranti, Giovani Meyrza Oka Putra Caesar, Jumaryoto Jumaryoto, Adeyinka Oye Akintunde, Bima Putra Pratama, Riza Zainuddin Ahmad, Wasito Wasito, Saifur Rehman

**Affiliations:** 1 Program Study of Animal Husbandry, Faculty of Agriculture, Universitas Sriwijaya, Palembang, South Sumatera, Indonesia.; 2 Department of Veterinary Reproduction, Universitas Airlangga, Surabaya, East Java, Indonesia.; 3 Research Center for Veterinary Science, National Research and Innovation Agency (BRIN), Bogor, West Java, Indonesia.; 4 Doctoral Program of Veterinary Science, Faculty of Veterinary Medicine, Universitas Airlangga, Surabaya, East Java, Indonesia.; 5 Department of Reproduction and Obstetrics, Faculty of Veterinary Medicine, Universitas Gadjah Mada, Sleman, Daerah Istimewa Yogyakarta, Indonesia.; 6 Faculty of Agroindustry, Department of Animal Husbandry, Universitas Mercu Buana Yogyakarta, Daerah Istimewa Yogyakarta, Indonesia.; 7 Directorate General of Animal Husbandry and Animal Health, Ministry of Agriculture of the Republic of Indonesia, Indonesia.; 8 Artificial Insemination Center, Singosari, Malang, East Java, Indonesia.; 9 Department of Agriculture and Industrial Technology, Babcock University, Ilishan-Remo, Ogun State, Nigeria.; 10 Research Center for Process Technology, National Research and Innovation Agency (BRIN), Serpong, South Tangerang, Banten, Indonesia.; 11 Department of Pathobiology, Faculty of Veterinary and Animal Sciences, Gomal University, RV9W+GVJ, Indus HWY, Dera Ismail Khan 27000, Pakistan.

**Keywords:** artificial insemination, cattle reproduction, delayed ovulation, fertility, follicular development, ovarian physiology, sustainable animal production, ultrasonography

## INTRODUCTION

Reproductive efficiency plays a crucial role in successful cattle production for both dairy and beef industries. Accurate ovulation timing is a key component of the reproductive cycle because it directly influences the likelihood of fertilization, successful conception, and optimal calving intervals [1]. Globally, delayed ovulation affects approximately 15%–25% of postpartum cows, with higher prevalence reported in high-producing dairy herds and in extensive production systems in Europe, North America, and parts of Asia [2]. Regionally, prevalence varies widely depending on herd management, nutrition, and environmental conditions.

Ovulation irregularities, particularly delayed ovulation, can significantly impair reproductive performance and productivity in cattle [3]. This condition is often difficult to detect clinically, as estrus signs may persist despite delayed ovulation. Delayed ovulation differs from general fertility decline, which may result from multifactorial issues including poor nutrition, infections, or herd management inefficiencies. It is also distinct from cystic ovarian disease (COD) or anovulation, which involve complete absence of ovulation or persistent cyst formation [4]. Therefore, a comprehensive understanding of the physiological mechanisms and factors underlying these disorders is essential.

Delayed ovulation refers to ovulation that occurs later than the normal timeframe after estrus, as opposed to anovulation, which is characterized by the complete absence of ovulation within a single estrous cycle [5]. In cattle, ovulation typically occurs approximately 24–32 h after peak estrus signs [6]. When ovulation is delayed, it can occur several hours or even days later, potentially disrupting the synchrony between ovum release and sperm presence [7]. This asynchrony can reduce the chances of optimal fertilization [2]. Understanding the difference between delayed ovulation and anovulation is crucial, as the management approaches and reproductive interventions required for each condition differ [8].

Delayed ovulation has a significant impact on reproductive performance. One of the main consequences is decreased conception rates due to the mismatch between the timing of ovulation and insemination [9]. Furthermore, calving intervals can be lengthened because of delayed recovery of the reproductive cycle, thereby reducing annual production output [10]. Economic losses associated with delayed ovulation include additional insemination costs, extended calving intervals, reduced milk yield, and delayed generation turnover. Estimated losses per affected cow range from USD 50 to 150 per cycle, depending on herd size and production system [2]. Delayed ovulation is a major contributor to repeat breeding syndrome in cattle, which increases costs for farmers due to repeated inseminations and additional reproductive management [11]. The cumulative impact of these disruptions affects not only individual animal fertility but also the farm’s overall economic efficiency [12].

Although the clinical and economic impacts of delayed ovulation are well understood, scientific publications that comprehensively outline its physiological mechanisms, risk factors, and effective management approaches remain relatively limited [13]. Numerous studies indicate that this condition is influenced by various factors, including nutritional adequacy, hormonal regulation, environmental stress, postpartum health conditions, and genetic factors [14–16]. However, the available scientific evidence is often fragmented and limited to specific populations or contexts. Recent research developments have shown increasing use of technologies such as ultrasonography, hormone profile analysis, and sensor-based estrus detection devices to improve the accuracy of identifying ovulatory disorders [17]. Despite these advances, existing reviews tend to focus either on physiological aspects or on management strategies, without fully integrating the multifactorial causes, diagnostic approaches, and practical reproductive interventions into a single comprehensive overview. Moreover, the implications of delayed ovulation across diverse production systems, as well as the cost-effectiveness of various management practices, are rarely addressed in depth. This lack of a dedicated, standalone synthesis that treats delayed ovulation as a distinct reproductive disorder, separate from general fertility decline, anovulation, or COD, highlights a critical knowledge gap and underscores the need for targeted guidance for veterinarians and livestock practitioners [18].

Despite growing recognition of delayed ovulation as a clinically relevant reproductive disorder, significant gaps persist in the scientific literature. Most studies address isolated aspects such as specific risk factors or diagnostic tools, with limited integration of physiological mechanisms, epidemiological data across production systems, and evidence-based management strategies. There is a notable scarcity of reviews that synthesize cross-disciplinary findings, evaluate the cost-effectiveness of interventions, or provide practical, system-specific recommendations. Furthermore, the long-term impacts on herd productivity, genetic progress, and sustainable cattle farming under varying climatic and management conditions remain insufficiently explored. This fragmen-tation limits the ability of researchers, veterinarians, and producers to implement precise, evidence-based interventions.

The present review aims to provide a comprehensive, integrative synthesis of delayed ovulation in cattle. It encompasses the underlying physiological mechanisms, multifactorial triggering factors, consequences for reproductive performance and farm economics, current diagnostic methods, and available management strategies. By critically analyzing and consolidating current findings from diverse production systems, this review seeks to bridge existing knowledge gaps, highlight practical implications, and identify priority areas for future research. Special emphasis is placed on cross-disciplinary insights to support the development of more precise, sustainable, and evidence-based reproductive management approaches for both dairy and beef cattle production.

## REVIEW METHODOLOGY

### Study design and review approach

This study was conducted as a narrative, integrative review to synthesize current scientific evidence on delayed ovulation in cattle, with a particular focus on its physiological mechanisms, etiological and risk factors, diagnostic approaches, clinical manifestations, and management strategies. An integrative approach was selected to incorporate evidence from diverse research methodologies, including experimental and observational studies, clinical trials, and applied field research.

The review was designed to integrate knowledge from multiple disciplines, including reproductive physiology, endocrinology, nutrition, animal management, and veterinary clinical science, in order to provide a comprehensive and practice-oriented perspective. Rather than performing a quantitative meta-analysis, this narrative synthesis emphasizes biological plausibility, clinical relevance, and applicability across different production systems (dairy and beef cattle). This approach is appropriate given the heterogeneity of study designs, outcome measures, and management contexts reported in the literature on delayed ovulation.

### Literature search strategy

A structured literature search was conducted using major scientific databases, including PubMed, Scopus, and Web of Science, supplemented by Google Scholar to capture relevant gray literature and highly cited publications. The search covered articles published between 2000 and 2025, reflecting both foundational studies and recent advances in reproductive management and diagnostic technologies.

**Search terms were used in various combinations and included:** “delayed ovulation,” “ovulatory disorders,” “follicular persistence,” “LH surge,” “postpartum ovulation,” “cattle reproduction,” “dairy cows,” “beef cattle,” “negative energy balance,” “endocrine disorders,” and “reproductive management.” Boolean operators were applied to refine the search and ensure comprehensive coverage of relevant topics.

### Study selection and data synthesis

Articles were screened based on their relevance to delayed ovulation in cattle, with inclusion criteria encompassing studies that addressed at least one of the following aspects: (i) physiological regulation of ovulation, (ii) nutritional, environmental, animal-related, endocrine, or infectious risk factors, (iii) diagnostic tools and criteria for ovulatory disorders, or (iv) management and therapeutic interventions aimed at improving ovulation timing and fertility.

Only peer-reviewed articles published in English were included. Studies focusing exclusively on other species or on reproductive disorders unrelated to ovulatory timing were excluded. After initial screening of titles and abstracts, full-text articles were reviewed to extract relevant data and conceptual insights.

Findings were synthesized narratively and organized thematically to highlight mechanistic pathways, clinical implications, diagnostic challenges, and management decision points. Particular attention was given to identifying knowledge gaps, inconsistencies among studies, and areas requiring further research to inform both clinical practice and future investigative priorities.

## PHYSIOLOGICAL BASIS OF OVULATION IN CATTLE

Ovulation in cattle is a complex physiological process that relies on the coordinated action of the hypothalamic-pituitary-ovarian (HPO) axis [19]. The hypothalamus releases gonadotropin-releasing hormone (GnRH) in a pulsatile manner, which is modulated not only by kisspeptin neurons, linking metabolic and environmental cues to GnRH secretion, but also by upstream neuroendocrine modulators such as neurokinin B and dynorphin, which fine-tune GnRH release [20].

**Figure 1:**
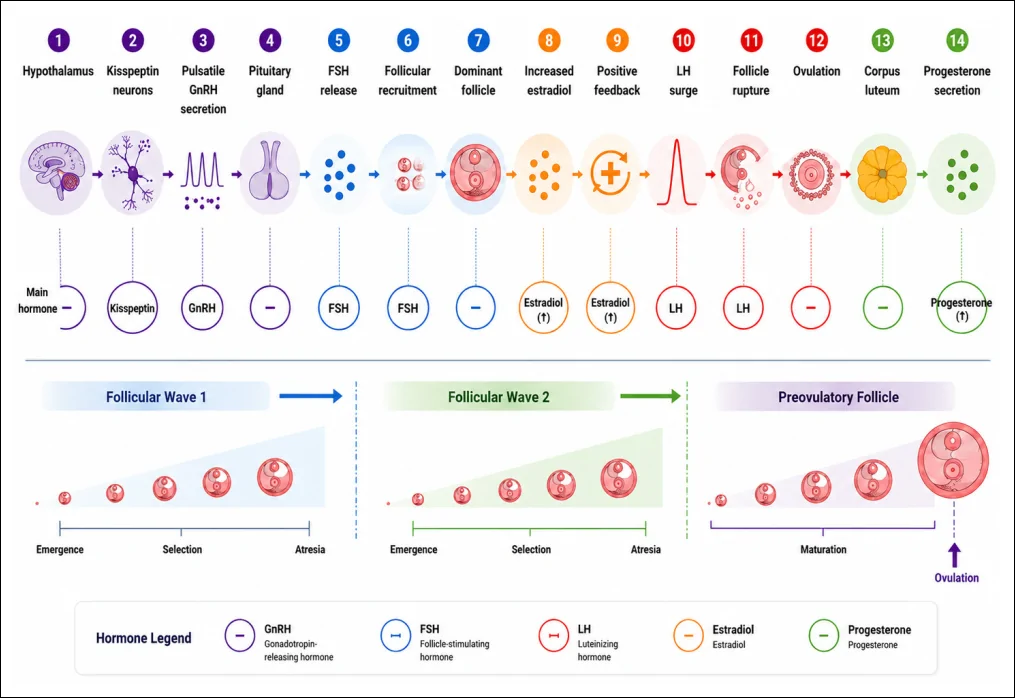
Hormonal regulation and follicular dynamics underlying ovulation in cattle. The figure was prepared by the authors based on information synthesized from previous studies describing the hypothalamic-pituitary-ovarian axis, gonadotropin secretion, follicular wave dynamics, endocrine regulation, and ovulatory mechanisms [19–30]. The graphical illustration was generated with the assistance of ChatGPT (OpenAI, San Francisco, CA, USA) and designed using Canva (Canva Pty Ltd., Sydney, Australia). The final figure was reviewed, refined, and validated by the authors to ensure scientific accuracy and consistency with the cited literature.

The rise in follicle-stimulating hormone (FSH) levels triggers the recruitment and growth of clusters of ovarian follicles, but only one dominant follicle develops into the preovulatory phase [21]. This dominant follicle produces increasing amounts of estradiol, and when estradiol peaks, a positive feedback mechanism activates the hypothalamus and pituitary to induce the luteinizing hormone (LH) surge, a critical signal for ovulation [22]. Figure 1 summarizes the coordinated hormonal regulation governing normal ovulation in cattle.

Follicular development occurs in several waves during each estrous cycle, characterized by recruitment, selection, and dominance [23, 24]. Ovulation is restricted to the wave coinciding with luteolysis, when declining progesterone levels increase pituitary responsiveness to estrogen [25]. Under normal conditions, the LH surge triggers molecular changes in the follicle, including activation of proteolytic enzymes and remodeling of the follicular wall, culminating in oocyte release [26].

Breed and age influence HPO axis sensitivity; high-producing dairy breeds often display altered LH surge amplitude and timing compared with beef or dual-purpose breeds, while older cows exhibit reduced follicular responsiveness to gonadotropins, potentially prolonging the preovulatory phase [27, 28].

Delayed ovulation occurs when hormonal regulation or follicular dynamics are disrupted. Mechanisms include residual high progesterone from incomplete luteolysis, impaired estradiol synthesis, or decreased LH receptor sensitivity, all of which can extend the preovulatory period [29]. Management and environmental factors, such as stress, metabolic disorders, and nutritional imbalances, can disrupt pulsatile GnRH release, thereby delaying ovulation [30].

## PATHOPHYSIOLOGY OF DELAYED OVULATION

Delayed ovulation in cattle occurs when oocyte release takes place later than the normal physiological timeframe, generally due to disturbances in endocrine regulation and ovarian activity [11]. Normally, ovulation is triggered by an LH surge that occurs in response to increased estradiol produced by the dominant follicle [31].

**The mechanistic flow can be summarized as:** cause → endocrine disruption → ovarian outcome → fertility consequence [32]. Primary triggers include stress, metabolic disorders, nutritional imbalances, and postpartum recovery delays, which disrupt pulsatile GnRH release from the hypothalamus. This leads to altered LH pulse frequency and amplitude, impairing the preovulatory LH surge and delaying oocyte maturation and follicle rupture [33, 34].

A delay in the LH surge can result in follicular persistence, where the dominant follicle remains unovulated despite large size and high steroidogenic activity [8]. Persistent follicles exhibit reduced estradiol production and impaired granulosa cell differentiation, decreasing responsiveness to gonadotropins and prolonging the follicular phase [35].

Oxidative stress further contributes by disrupting steroidogenesis and LH receptor signaling, suppressing the LH surge [36]. Low LH receptor expression in granulosa and theca cells or impaired luteotropic response compromises final follicle maturation [37].

**Temporary versus recurrent delayed ovulation is mechanistically distinct:** transient delays typically arise from postpartum recovery or acute stress, whereas recurrent delays are often due to chronic metabolic, endocrine, or inflammatory disorders [38].

Delayed ovulation is also linked to impaired corpus luteum (CL) function, particularly when CLs arise from persistent follicles [39]. These CLs produce less progesterone and have shortened functional lifespan, resulting in prolonged inter-estrus intervals, increased repeat breeding, and reduced conception rates [40, 41].

Neuroendocrine interactions via the hypothalamic-pituitary-adrenal (HPA) axis exacerbate delays [42]. Elevated cortisol from stress or metabolic challenges suppresses GnRH pulsatility, reduces LH production, and directly impairs ovarian steroidogenic enzymes and gonadotropin receptor expression [43, 44].

**Figure 2:**
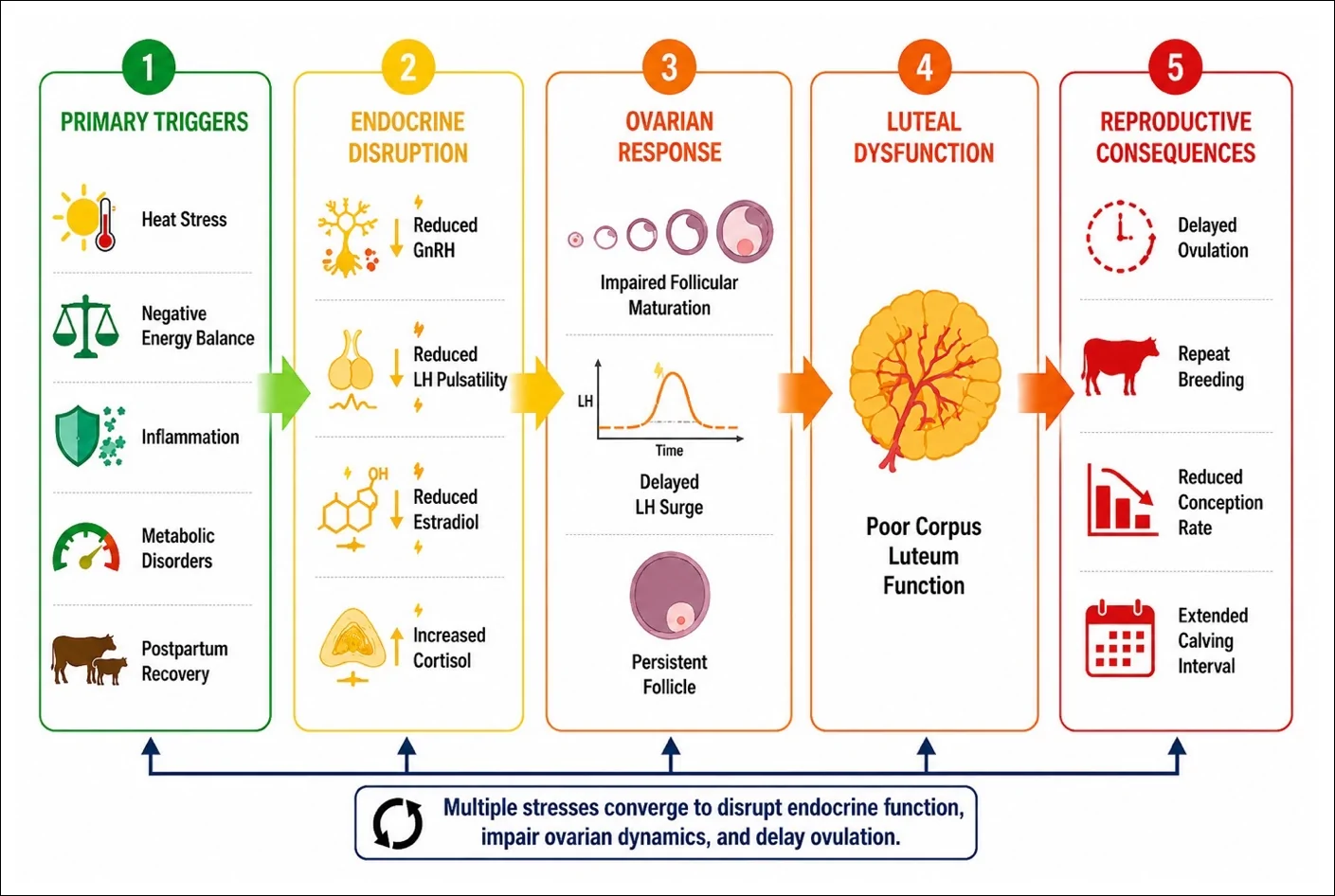
Integrated pathophysiological mechanisms of delayed ovulation in cattle. The figure was prepared by the authors based on information synthesized from previous studies [8, 11, 31, 32, 33, 34, 35, 36, 37, 38, 39, 40, 41, 42, 43, 44]. The graphical illustration was generated with the assistance of ChatGPT (OpenAI, San Francisco, CA, USA) and designed using Canva (Canva Pty Ltd., Sydney, Australia). The final figure was critically reviewed, refined, and validated by the authors to ensure scientific accuracy and consistency with the cited literature.

Figure 2 illustrates the integrated pathophysiological cascade leading to delayed ovulation. 

## ETIOLOGICAL FACTORS

Delayed ovulation in cattle is a condition triggered by various factors, including nutrition, environment, the animal's physiological condition, endocrine–metabolic disorders, and the presence of infections or inflammatory processes [45]. Rather than acting in isolation, these factors often interact, whereby nutritional deficiencies can exacerbate endocrine imbalances, environmental stressors may amplify inflammatory responses, and physiological condition influences susceptibility to both metabolic and infectious triggers.

A comprehensive understanding of these interconnected triggers is essential for developing effective prevention and management strategies to improve reproductive efficiency [46]. Table 1 presents a summary of the etiologic factors contributing to delayed ovulation in cattle.

## NUTRITIONAL FACTORS

Nutritional factors play a crucial role in regulating reproductive function in cattle and are a key determinant of delayed ovulation [47]. Nutritional disturbances, including energy deficiency or micronutrient imbalance, modulate endocrine function, ovarian steroid metabolism, and gonadotropic activity, thereby inhibiting normal ovulation [48].

Negative energy balance (NEB) is a primary cause of delayed ovulation, particularly in dairy cows during early lactation [49]. NEB lowers blood levels of glucose, insulin, and Insulin-like growth factor-1 (IGF-1), which in turn reduces ovarian sensitivity to gonadotropin stimulation [50]. Circulating IGF-1 concentrations below approximately 80–100 ng/mL are associated with reduced follicular growth and higher risk of delayed ovulation, as low IGF-1 inhibits granulosa cell proliferation and decreases LH receptor expression in the dominant follicle [51]. Consequently, the preovulatory follicle may fail to respond optimally to the LH surge, leading to delayed ovulation or the formation of persistent follicles [52].

Excessive lipid mobilization during NEB produces non-esterified fatty acids (NEFA) and β-hydroxybutyrate (BHB). Plasma NEFA levels >0.4–0.6 mmol/L and BHB >1.2–1.4 mmol/L during early lactation indicate subclinical ketosis, which is toxic to ovarian cells and impairs steroidogenesis [53]. In mid-lactation cows, NEB is generally less severe, but ongoing high milk production with inadequate dietary energy can still elevate NEFA and BHB, contributing to delayed ovulation.

**Table 1:** Etiological factors of delayed ovulation in cattle.

**Factor category**	**Sub-factor**	**Mechanism / Impact on ovulation**	**References**
Nutrition	Negative Energy Balance (NEB)	Reduced glucose, insulin, and IGF-1 → decreased ovarian sensitivity to gonadotropins → impaired dominant follicle maturation → delayed ovulation or persistent follicles. Elevated non-esterified fatty acids (NEFA) (>0.4–0.6 mmol/L) and BHB (>1.2–1.4 mmol/L) cause follicular toxicity.	[50, 53]
	Micronutrients (Se and Vit E)	Antioxidant deficiency → oxidative stress → impaired granulosa cell function and steroidogenesis → disrupted LH surge → delayed ovulation.	[55, 56]
	Vitamin A	Impaired granulosa cell differentiation and post-ovulatory progesterone synthesis → affected oocyte maturation → prolonged follicular phase.	[57]
	Body Condition Score (BCS)	Low BCS: suppressed GnRH/LH activity → delayed follicle response; High BCS: insulin resistance and systemic inflammation → follicle asynchrony → delayed ovulation.	[58–60]
Environment and Management	Heat stress	Elevated cortisol → decreased GnRH/LH pulsatility → impaired steroidogenesis and oocyte maturation → delayed ovulation or persistent follicles.	[62–65]
	Mistimed estrus detection & insemination	Follicle-LH surge asynchrony → delayed or missed ovulation → reduced conception.	[66–68]
	High production intensity	Increased metabolic demand → NEB and metabolic stress → impaired follicular steroidogenesis and endocrine function → delayed ovulation.	[70–72]
	Housing / confinement stress	Restricted movement, poor ventilation, and heat retention → endocrine disruption → follicle growth delays → delayed ovulation.	[73]
Animal-related factors	Parity	Primiparous cows: higher NEB and immature metabolic capacity → lower GnRH/LH pulsatility → increased risk of delayed ovulation.	[76–78]
	Breed	High-yielding dairy breeds more susceptible than beef or indigenous breeds due to higher metabolic demands and variability in LH receptor expression → altered follicular dynamics.	[78, 79]
	Postpartum period	NEB + incomplete uterine involution → subclinical inflammation → suppressed GnRH/LH → delayed ovulation.	[82–84]
Endocrine and metabolic disorders	Hypothyroidism	Low T3/T4 → reduced IGF-1 → slow follicle growth → weakened LH surge → delayed ovulation.	[86–88]
	Hyperprolactinemia	High prolactin → suppressed GnRH/LH pulsatility → impaired granulosa cell response → delayed ovulation.	[90–93]
	Insulin resistance and metabolic stress	Disrupted insulin signaling → impaired steroidogenesis → poor oocyte quality → persistent follicles → delayed ovulation.	[94–99]
Infection and inflammation	Subclinical endometritis	Increased IL-1β, IL-6, TNF-α → suppressed GnRH/LH → delayed follicle maturation → persistent follicles.	[102–105]
	Viral infections (infectious bovine rhinotracheitis and bovine viral diarrhea)	Ovarian damage + altered hormone profile → impaired follicle development → delayed ovulation.	[107–110]
	Systemic inflammation	Elevated cortisol and cytokines → decreased GnRH/LH and granulosa cell apoptosis → impaired oocyte maturation → delayed ovulation.	[112–115]

Micronutrients play a crucial role in maintaining optimal reproductive function [54]. Selenium and vitamin E act as antioxidants, protecting ovarian tissue from oxidative stress and supporting enzymes in steroidogenesis [55]. Dietary selenium is supplied via forages, cereal grains, and premixes at 0.1–0.3 mg Se/kg dry matter, while vitamin E is commonly given at 500–1,000 IU/day during the periparturient period. Deficiencies impair follicular maturation and estradiol production, disrupting the positive feedback necessary to trigger the LH surge [56]. Vitamin A, primarily from β-carotene in green forages or supplemented at 50,000–100,000 IU/day, supports granulosa cell differentiation, follicle development, and post-ovulatory progesterone synthesis; deficiency prolongs the follicular phase and inhibits oocyte maturation [57].

Body condition score (BCS) provides an integrated measure of energy status [58]. Low BCS suppresses GnRH and LH activity, prolonging the time to ovulation, whereas excessively high BCS can induce insulin resistance and systemic inflammation, thereby negatively affecting ovarian function and oocyte quality [59]. Changes in BCS during the transition period are particularly informative: cows losing >0.5 BCS units in the first 4–6 weeks postpartum experience greater metabolic stress and hormonal imbalance, increasing delayed ovulation risk compared with cows maintaining a stable BCS, even if their absolute BCS is adequate [60].

## ENVIRONMENTAL AND MANAGEMENT FACTORS

Environmental factors and husbandry management play a significant role in regulating cow reproductive function, particularly regarding follicle dynamics and the timing of ovulation [61]. One of the most influential environmental factors is heat stress, which occurs when ambient temperatures exceed a cow’s ability to maintain stable body temperature [62]. Heat stress is often quantified using the temperature–humidity index (THI), with values above 68–72 indicating the onset of heat stress and values >78 associated with severe heat stress and increased risk of delayed ovulation.

Heat stress triggers increased glucocorticoid production, particularly cortisol, which suppresses GnRH pulse frequency and LH release amplitude, weakening or delaying the LH surge required for ovulation [28, 63]. Additionally, heat stress disrupts granulosa cell steroidogenesis, reduces estradiol production, and impairs oocyte maturation [64]. Cows exposed to sustained temperature–humidity index above critical thresholds during the peri-estrus period exhibit a higher incidence of delayed ovulation and persistent follicles, reflecting combined endocrine and cellular effects of thermal stress [65].

From a management perspective, errors in estrus detection and insemination timing are significant contributors to delayed ovulation [66]. Misidentifying estrus phases can create a mismatch between peak follicle development and insemination timing. In synchronization programs, improper administration of prostaglandin or GnRH, or mistimed insemination, can disrupt preovulatory follicle development [67, 68]. Real-world examples include delayed or missed GnRH injection in Ovsynch protocols, insemination before the dominant follicle reaches ovulatory size, or failure to administer prostaglandin at recommended intervals. These errors prolong the follicular phase, inhibit the LH surge, and increase the risk of delayed or absent ovulation [69].

Production pressure, particularly in high-yielding dairy cows, also contributes to ovulatory disorders [2]. High-producing cows face elevated metabolic demands, predisposing them to NEB [70], which not only reduces nutritional adequacy but also acts as a metabolic stressor suppressing reproductive function [71]. Efficiency of ovarian tissue energy utilization decreases, and levels of IGF-1, insulin, and other metabolic hormones that support follicular response decline [72].

**Housing systems further modulate these effects:** cows in tie-stall systems often have restricted movement, reduced heat dissipation, and less overt estrus expression compared with free-stall systems, which provide better ventilation, cow comfort, and behavioral estrus expression. In beef cattle, lower production intensity generally reduces the risk of ovulatory disorders; however, management factors such as feeding inconsistencies, transport stress, high stocking density, or inadequate thermal comfort can still disrupt ovarian function [73].

## ANIMAL-RELATED FACTORS

Factors directly related to the individual animal play a significant role in regulating reproductive function and significantly influence the occurrence of delayed ovulation in cows [74]. Variations in age, parity, breed type, and postpartum physiological condition influence hormonal responses, follicle development, and reproductive recovery rates [75].

Parity is a major factor influencing ovarian function [76]. Cows can be broadly categorized as heifers (young primiparous cows, first parity, ≤3 years of age) and multiparous cows (second parity and above, >3 years of age). Primiparous cows show a higher incidence of delayed ovulation, primarily due to competition between somatic growth and milk production for energy resources [77]. In young cows, the metabolic capacity to support ovarian function is not yet fully optimized, making NEB more likely, resulting in decreased GnRH and LH pulsations [78]. Multiparous cows generally exhibit more consistent follicular responses to gonadotropins and a more favorable metabolic hormone profile, thereby reducing the risk of delayed ovulation [1].

Age-related reproductive changes also influence ovarian responsiveness. As cows age beyond 6–7 years, there is evidence of reduced follicular reserve, lower granulosa cell proliferation, and decreased sensitivity to gonadotropins, all of which can prolong the preovulatory phase and increase the likelihood of ovulatory delay [74].

Differences in cattle breeds also influence reproductive sensitivity to environmental and nutritional conditions [46]. High-yielding dairy cattle, such as Holsteins (*Bos taurus*), are more susceptible to delayed ovulation than dual-purpose or local breeds, due to their high metabolic demands, which affect energy status and metabolic hormones such as insulin and IGF-1 [79]. Conversely, beef and indigenous breeds, including Zebu-derived cattle (*Bos indicus*), typically exhibit better reproductive resilience and tolerance to nutritional and thermal stress [80]. These differences are partly related to genetic variation in reproductive and metabolic pathways, including gonadotropin signaling (*LHCGR* and *FSHR*), steroidogenesis (*CYP19A1* and Steroidogenic acute regulatory protein [*STAR*]), and energy metabolism (*Leptin* and *IGF1*); however, current genetic markers remain limited in predicting ovulation outcomes, and caution is required to avoid overinterpretation [81]. Comparative studies have shown that *Bos indicus* cattle often maintain more stable LH pulsatility and follicular growth under heat stress compared with *Bos taurus* breeds, reducing the risk of ovulatory delay.

The postpartum period is a critical phase that significantly influences the accuracy of ovulation [82]. During this period, cows experience significant metabolic changes due to the onset of milk production, accompanied by decreased reproductive hormone levels and increased energy requirements [83]. NEB in early lactation suppresses LH pulse frequency, inhibiting preovulatory follicle maturation and prolonging the interval to first postpartum ovulation [8]. Furthermore, incomplete uterine involution can trigger subclinical inflammation and increase prostaglandin production, which disrupts follicle development and CL function [36]. Subclinical endometritis activates inflammatory cytokines (e.g., tumor necrosis factor-alpha [TNF-α] and Interleukin-1 beta [IL-1β]), which suppress GnRH secretion via neuroendocrine pathways, increasing delayed ovulation risk [84].

## ENDOCRINE AND METABOLIC DISORDERS

Hormonal and metabolic disorders are major factors that can disrupt the function of the HPO axis and play a significant role in delayed ovulation in cows [3]. These disorders include hypothyroidism, hyperprolactinemia, insulin resistance, and general metabolic stress, all of which can alter gonadotropin production, follicle development, and ovarian responsiveness to the LH surge [85].

Hypothyroidism decreases basal metabolic rate, alters reproductive hormone regulation, and reduces ovarian sensitivity to gonadotropins [86]. Clinically, it is characterized by low circulating Triiodothyronine and Thyroxine, often accompanied by lethargy, reduced milk yield, and poor reproductive performance. Low thyroid hormone levels decrease IGF-1 production, impair follicle growth, and reduce steroidogenesis, preventing estradiol from reaching the threshold needed to trigger a robust LH surge [87, 88]. Hypothyroidism may also increase thyrotropin-releasing hormone, stimulating prolactin release and further disrupting the estrous cycle [89].

Hyperprolactinemia directly suppresses GnRH secretion [90]. Elevated prolactin reduces LH pulse frequency, impairs granulosa cell responsiveness to FSH, and inhibits oocyte maturation [91]. It also decreases estradiol synthesis by interfering with steroidogenic enzymes [92]. It is important to distinguish physiological postpartum hyperprolactinemia, transient elevations in early lactation supporting milk production, from pathological hyperprolactinemia, where persistently high prolactin outside the normal postpartum period disrupts GnRH/LH secretion and ovulation. Failure to reach estradiol thresholds prevents the LH surge, making pathological hyperprolactinemia a recognized contributor to delayed ovulation and anestrus [93].

Insulin resistance and metabolic stress impair ovarian function by reducing insulin signaling efficacy, which disrupts follicle development and estradiol synthesis through IGF-1 interactions [59, 94, 95]. Clinically, insulin resistance is often associated with overconditioned cows, elevated circulating insulin or glucose, reduced insulin sensitivity in early lactation, and poor reproductive responses despite adequate nutrition [60]. This condition limits energy availability to the ovaries and decreases follicular response to LH, contributing to delayed ovulation [96].

Metabolic stress, including NEB in early lactation, reduces blood IGF-1, glucose, and insulin [97]. This suppresses GnRH secretion, lowers LH pulse amplitude, and slows dominant follicle growth [98]. Elevated lipid metabolites such as NEFA and BHB can be toxic to granulosa cells and oocytes, impairing follicle quality and promoting persistent follicles [99]. Collectively, these endocrine and metabolic disruptions delay ovulation and impair functional CL formation [100].

## INFECTIOUS AND INFLAMMATORY CAUSES

Infection and inflammation, both local and systemic in the reproductive tract, are important factors that can reduce ovarian function and trigger delayed ovulation in cows [101]. These mechanisms act through negative effects on the HPO axis, elevated proinflammatory cytokines, and direct disruption of follicular development and oocyte quality [91].

Subclinical endometritis is a major cause of ovulatory dysfunction in postpartum cows [102]. Although asymptomatic, this condition is characterized by inflammatory cell infiltration of the uterine mucosa and increased cytokines such as IL-1β, Interleukin-6 (IL-6), and TNF-α [103]. Diagnostic thresholds are commonly defined by polymorphonuclear neutrophil (PMN) proportions: ≥18–20% PMN at 21–33 days postpartum and ≥5–10% PMN after 34–47 days postpartum, depending on sampling method and herd conditions [103]. These cytokines can enter the bloodstream, inhibit GnRH activity in the hypothalamus, reduce the frequency of LH pulses, and disrupt granulosa cell proliferation in the dominant follicle [104]. Subclinical endometritis also alters the uterine environment, increasing prostaglandin production, which affects ovarian function and can inhibit post-ovulatory CL formation [105]. Together, these effects prolong the follicular phase and increase delayed ovulation risk [106].

Viral reproductive diseases, particularly infectious bovine rhinotracheitis (IBR) and bovine viral diarrhea (BVD), significantly impact estrous cyclicity and ovulation [107]. IBR can cause oophoritis and granulosa cell necrosis, disrupting preovulatory follicle development [108], whereas BVD causes immunosuppression and decreases ovarian sensitivity to FSH and LH [109]. Acute BVD infection also reduces estradiol levels and impairs the positive feedback necessary to trigger the LH surge [110]. Effective herd vaccination programs mitigate these effects by reducing viral circulation, systemic inflammation, and the likelihood of ovulatory disruption [111].

Systemic inflammation from mastitis, clinical metritis, pneumonia, or metabolic stress also contributes to delayed ovulation [112, 113]. Elevated cortisol suppresses GnRH secretion, decreases LH pulse amplitude, and impairs ovarian steroidogenic enzyme activity [114]. Inflammatory cytokines can induce granulosa cell apoptosis, inhibit estradiol synthesis, and disrupt oocyte–granulosa communication, impairing oocyte maturation [115]. Timely antimicrobial or anti-inflammatory interventions, particularly during the early postpartum period, are critical for limiting uterine and systemic inflammation, preserving LH surge dynamics, and preventing delayed ovulation [2].

Preventive and herd level control strategies include maintaining strict vaccination protocols, monitoring for subclinical uterine infections, timely postpartum interventions, and management practices that minimize stress and inflammation. These measures reduce systemic and local inflammatory challenges, thereby supporting normal HPO axis function and timely ovulation [112].

**Figure 3:**
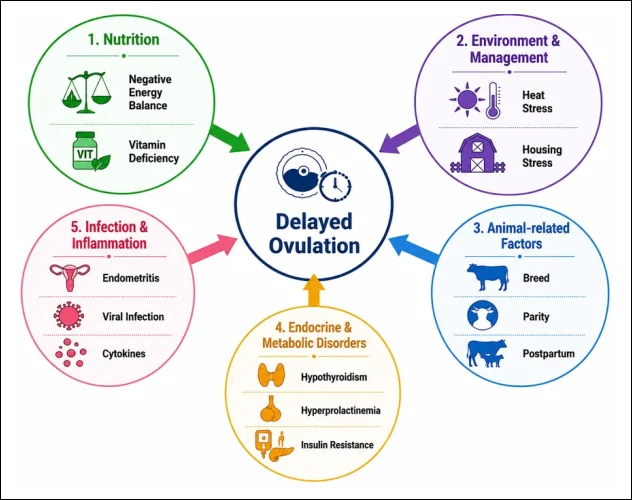
Multifactorial risk factors contributing to delayed ovulation in cattle. The figure was prepared by the authors based on information synthesized from previous studies [2, 91, 101, 102, 103, 104, 105, 106, 107, 108, 109, 110, 111, 112, 113, 114, 115]. The graphical illustration was generated with the assistance of ChatGPT (OpenAI, San Francisco, CA, USA) and designed using Canva (Canva Pty Ltd., Sydney, Australia). The final figure was critically reviewed, refined, and validated by the authors to ensure scientific accuracy and consistency with the cited literature.

Figure 3 summarizes the major interacting risk factors associated with delayed ovulation in cattle. 

## CLINICAL MANIFESTATIONS AND REPRODUCTIVE IMPACTS

Delayed ovulation in cattle is a reproductive disorder characterized by delayed release of oocytes from preovulatory follicles, resulting in a variety of clinical manifestations at the individual and herd level [116]. A hallmark clinical sign is follicular persistence, in which the dominant follicle remains in the ovary without ovulation despite reaching physiological size [117]. Ultrasonography typically shows a large, unruptured follicle, sometimes with partial luteinization of the follicle wall [118]. This follicular persistence leads to asynchronous follicular waves and disrupts normal hormonal patterns [27]. Clinically, a practical diagnostic approach combines sequential estrus monitoring, ovarian ultrasonography to identify persistent follicles, and evaluation of metabolic or inflammatory risk factors to guide targeted hormonal or management interventions.

Delayed ovulation also affects estrus behavior [119]. Cows may show prolonged, irregular, or weak estrus (silent heat) [120], caused by an imbalance of estradiol and progesterone due to impaired follicle dynamics and CL formation [11]. Weak estrus expression reduces the accuracy of estrus detection in the field, contributing to mistimed insemination [45].

At the reproductive level, delayed ovulation decreases conception rates [3]. The condition causes a mismatch between available healthy oocytes and optimally capacitated sperm [121], reducing fertilization success and increasing early embryonic loss [122]. It often results in repeat breeder status, defined as failure to conceive after three or more consecutive inseminations in the absence of anatomical or infectious reproductive disorders. This increases reproductive costs, including additional inseminations, labor, and reduced milk or meat production [123].

Long-term consequences include prolonged calving intervals—the time between successive births [10]. At the herd level, these effects differ by production system: in dairy herds, prolonged calving intervals reduce annual milk yield, increase days open, and raise replacement rates; in beef herds, consequences include lower calf crop percentages, delayed weaning weights, and slower herd turnover [124]. Chronic ovarian dysfunction may also lead to luteal insufficiency, disrupting subsequent estrus cycles and reducing overall herd reproductive efficiency [19].

## DIAGNOSTIC APPROACHES

Detecting and diagnosing delayed ovulation in cattle requires a comprehensive approach, as the condition is often subclinical and difficult to recognize through behavioral observations alone [120]. Various diagnostic methods are available, ranging from ultrasonography and hormone profiling to behavioral estrus detection technologies and molecular biomarkers [125]. It is important to differentiate between research-grade diagnostics, which provide detailed mechanistic insights but require laboratory infrastructure, and field-applicable tools that are feasible, rapid, and cost-effective for routine herd management. Table 2 presents a summary of diagnostic methods used to detect delayed ovulation in cattle.

**Table 2:** Diagnostic approaches for delayed ovulation in cattle.

**Diagnostic method**	**Principle / Mechanism**	**Application / Notes**	**References**
Ultrasonography	Non-invasive imaging to monitor ovarian and follicular dynamics	Serial scanning of dominant follicles (≥18–25 mm) to assess growth, persistence, regression, and ovulation; detection of delayed corpus hemorrhagicum or partial luteinization indicates delayed ovulation. Highly effective but requires skilled operator and equipment.	[126–131]
Hormonal profiling	Measurement of reproductive hormone levels (LH and progesterone)	LH surge detection indicates ovulation timing; progesterone measurement 5–7 days post-estrus confirms corpus luteum function. Provides mechanistic insight but requires laboratory infrastructure and frequent sampling; mainly used in research or advanced herd management.	[132–138]
Behavioral and estrus detection tools	Monitoring estrus-related behavior induced by estrogen	Field-applicable tools include activity sensors, pedometers, pressure mats, and visual observation (restlessness, vocalization, tail elevation, mucus discharge). Artificial insemination-based systems integrate activity, temperature, and historical cycle data to predict ovulation and flag cows at risk for delayed ovulation.	[139–145]
Molecular / Biomarker approaches	Analysis of inflammatory, metabolic, and follicular markers	IL-1β, IL-6, TNF-α reflect local/systemic inflammation; NEFA, BHB, glucose, and insulin indicate energy balance. Valuable for early detection and research; best used as complementary tools alongside ultrasonography and hormonal profiling. Not practical as standalone routine field diagnostics due to cost and infrastructure needs.	[146–156]

## ULTRASONOGRAPHY

Ultrasonography is a non-invasive imaging technique useful for assessing cattle reproductive function, particularly for monitoring follicle dynamics and ovulation timing [126]. Serial monitoring allows observation of dominant follicle growth during the follicular phase [127]. Delayed ovulation is suspected when a dominant follicle ≥18–25 mm persists for >48–72 h after expected ovulation or estrus without rupture. Other ultrasonographic indicators include increased follicular wall thickness, partial luteinization, and delayed corpus hemorrhagicum formation [128]. Serial scans at 12–24-h intervals enable reliable detection of ovulation by observing the disappearance of the pre-dehiscent follicle and the appearance of the corpus hemorrhagicum [129, 130]. While ultrasonography is highly effective in research and modern herd reproductive management, equipment costs and operator expertise can limit its use in small-scale or resource-limited farms [131].

## HORMONAL PROFILING

Hormonal measurements are critical for understanding reproductive dynamics, particularly LH surge timing and luteal function [132, 133]. Practical field interpretation requires frequent sampling during the peri-estrus period (every 2–4 h, 12–36 h after estrus onset) to detect the LH surge; failure to detect the peak may indicate delayed or absent ovulation [2, 134]. Progesterone measurements, optimally 5–7 days post-estrus or insemination, confirm CL formation [135–137]. Although hormonal profiling provides mechanistic insight, costs, laboratory infrastructure, and sample processing limit routine field application [138].

## BEHAVIORAL AND ESTRUS DETECTION TOOLS

Estrus detection remains critical for managing reproduction and determining insemination timing [139]. Physiologically, estrus is characterized by behavioral changes reflecting increased locomotor activity due to estrogen stimulation [140]. Activity monitors, pedometers, and pressure sensors can track these behaviors [17, 141–143]. In delayed ovulation, estrus behavior may occur without ovulation, highlighting the need to combine behavioral monitoring with ovarian or hormonal assessment [142]. Traditional visual observation remains practical for small-scale farms but depends on observer skill and frequency [144]. Artificial insemination (AI)-based estrus detection systems enhance accuracy by integrating activity, diet, body temperature, and historical cycle data, helping flag cows at risk for delayed ovulation [125, 145]. These field-applicable tools are more feasible and cost-effective than laboratory-based hormone or molecular assays.

## MOLECULAR OR BIOMARKER APPROACHES

Molecular biomarkers are increasingly applied in research to study delayed ovulation mechanisms [146]. Changes in ovarian function, local inflammation, and metabolic status are reflected in biomarkers such as IL-1β, IL-6, TNF-α, NEFA, BHB, IGF-1, and insulin [147, 148]. Ovulation involves a controlled inflammatory process, and disruptions in prostaglandins, interleukins, or TNF-α indicate impaired follicle rupture or systemic inflammation [149, 150]. Granulosa cell or follicular fluid analysis can reveal these molecular changes, making them important research indicators [151].

Metabolic biomarkers, including NEFA, BHB, glucose, and insulin, are also valuable for understanding energy balance and reproductive function [152, 153]. Prolonged NEB disrupts steroidogenesis and follicular development, increasing delayed ovulation risk [78, 154].

Despite their diagnostic potential, molecular and hormonal assays are limited by cost, need for laboratory infrastructure, and turnaround time [155]. Thus, their primary use is in research or as complementary tools alongside ultrasonography and clinical evaluation, rather than as standalone diagnostics in routine herd management [156].

## COMPARATIVE ANALYSIS WITH OTHER REPRODUCTIVE DISORDERS

Delayed ovulation in cattle is a reproductive disorder that must be differentiated from other ovarian disorders, such as COD, silent heat, and anovulation [157]. Clear differentiation is essential to avoid diagnostic errors and inappropriate treatments, which can negatively affect conception rates and reproductive efficiency [158]. Table 3 presents a comparative summary of delayed ovulation versus COD, silent heat, and anovulation, with interpretive commentary to guide clinical decision-making.

## DELAYED OVULATION VS COD

Although both involve ovulatory dysfunction, the pathophysiology and clinical manifestations of delayed ovulation and COD differ substantially. In delayed ovulation, the dominant follicle develops normally, but ovulation occurs later than expected, whereas in COD, abnormal follicles or cysts (typically >25 mm in diameter and persisting for more than 10 days) fail to ovulate and remain on the ovary [19]. COD is often associated with chronic LH dysregulation or reduced ovarian responsiveness, while delayed ovulation is typically due to transient LH surge attenuation caused by metabolic stress, inflammation, or suboptimal management [159].

Diagnostic overlap can occur if a large persistent follicle is observed during a single examination without serial ultrasonographic monitoring. Misclassification of delayed ovulation as COD may lead to inappropriate repeated hormonal treatments (e.g., GnRH or controlled internal drug release [CIDR]), further disrupting follicular dynamics and conception rates [160]. Serial monitoring of follicle development and luteal assessment is therefore critical to minimize misdiagnosis [160].

**Table 3:** Comparison of delayed ovulation with other reproductive disorders in cattle.

**Reproductive disorder**	**Pathophysiology/Mechanism**	**Clinical manifestation**	**Difference from delayed ovulation**	**References**
Cystic Ovarian Disease (COD)	Formation of abnormal follicle/cyst (>25 mm), chronic LH dysregulation, reduced ovarian responsiveness	Persistent cysts, abnormal or irregular estrus behavior; easily detected by palpation or ultrasonography	Delayed ovulation: dominant follicle develops normally but ovulates later than expected. COD: abnormal cysts persist and fail to ovulate; typically more chronic LH disruption.	[19, 159–160]
Silent Heat	Ovulation occurs at normal time with intact LH surge, but estrus behavior is suppressed	Absence of observable estrus signs (mounting, vocalization, vulvar edema)	Delayed ovulation involves true delay in ovulation due to attenuated or postponed LH surge; estrus behavior may still be present, prolonged, or irregular.	[28, 4]
Anovulation	Complete failure of ovulation due to chronic endocrine disorders or prolonged metabolic stress	No oocyte release and no corpus luteum formation across one or more cycles; persistently low progesterone	Delayed ovulation eventually results in ovulation. Anovulation shows total absence of ovulation; typically more chronic and severe.	[8, 161–163]

## DELAYED OVULATION VS SILENT HEAT

Silent heat is characterized by ovulation occurring at the normal physiological time in the absence of overt estrus behavior [28]. Endocrine events, including the LH surge, are normal; the absence of behavioral signs such as mounting, vocalization, or vulvar edema is the distinguishing feature [4].

In contrast, delayed ovulation involves endocrine disruption with a postponed or attenuated LH surge, resulting in delayed oocyte release, even if estrus behavior may be present, prolonged, or irregular [2]. Thus, silent heat primarily reflects altered estrus expression, whereas delayed ovulation reflects impaired ovulatory physiology. Failure to differentiate these can lead to inappropriate interventions: silent heat benefits from improved estrus detection (e.g., activity sensors, progesterone monitoring), while delayed ovulation requires targeted hormonal or metabolic therapy to restore normal ovulatory timing [17].

## DELAYED OVULATION VS ANOVULATION

Anovulation is a condition in which ovulation does not occur at all during one or more estrous cycles [8]. Unlike delayed ovulation, where ovulation eventually occurs, anovulation reflects total failure of oocyte release and CL formation [161]. The causes of anovulation are typically more chronic, including severe endocrine dysfunction, prolonged NEB, or persistent systemic disease [162].

Diagnosis relies on repeated absence of ovulation via serial ultrasonography and persistently low progesterone, whereas delayed ovulation is diagnosed by identifying a postponed ovulatory event following estrus or follicular dominance [163]. Misdiagnosing delayed ovulation as anovulation may lead to unnecessarily aggressive hormonal protocols and increased reproductive costs, underscoring the importance of accurate differentiation.

## MANAGEMENT AND TREATMENT STRATEGIES

Managing delayed ovulation in cattle requires a comprehensive approach, including hormonal therapy, nutritional management, stress and environmental management, postpartum care, and the use of assisted reproductive technologies (ART) [14]. These strategies aim to restore normal ovulatory cycles, increase conception rates, and maximize overall reproductive efficiency [164]. Table 4 summarizes the management and therapy strategies used to address delayed ovulation in cattle.

## HORMONAL THERAPIES

Hormonal therapy is the most commonly used method to treat delayed ovulation in cattle, particularly in postpartum dairy cows, which are susceptible to folliculogenesis and ovulatory response disorders [165]. Exogenous hormone administration aims to normalize endocrine function, stimulate follicle maturation, optimize the LH surge, and ensure ovulation occurs at the physiological time, thus supporting increased reproductive efficiency [166]. However, the choice of hormonal agent must consider follicular status, metabolic condition, and potential risks associated with repeated hormonal exposure. Effectiveness may vary significantly between herds depending on management, nutrition, and overall health status.

**Table 4:** Management and therapy strategies to overcome delayed ovulation in cattle.

**Strategy / Approach**	**Mechanism / Action**	**Application / Description**	**References**
Hormonal therapies	Stimulate ovulation through exogenous hormones	GnRH induces artificial LH surge in dominant follicles; hCG acts as LH agonist and promotes luteinization. Synchronization protocols (Ovsynch, G6G, Double Ovsynch) coordinate ovulation, improve follicle uniformity, and optimize AI timing. Choice depends on follicular status, metabolic condition, and herd management.	[165–175]
Nutritional management	Optimize energy status and micronutrient supply	Maintain ideal BCS 3.0–3.5; prevent or minimize NEB; supplement selenium, zinc, copper, manganese, vitamin E, and vitamin A. Supports follicle maturation, LH sensitivity, steroidogenesis, and overall ovarian function.	[176–186]
Environmental and stress management	Reduce environmental and stress effects on reproduction	Manage heat stress through improved ventilation, cooling systems, and shading; optimize stocking density, barn comfort, and lighting. Helps maintain hormonal balance, estrus expression, and ovulatory competence.	[187–195]
Postpartum management	Support postpartum recovery for timely ovulation	Implement uterine health programs to prevent endometritis/metritis; optimize transition cow management with BCS monitoring, balanced nutrition, and energy balance control. Enables early differentiation of physiological vs. pathological delayed ovulation and accelerates reproductive recovery.	[196–201]
Assisted reproduction technologies	Synchronize ovulation and improve AI timing	Fixed-time artificial insemination (FTAI) with hormonal protocols (GnRH + prostaglandins) standardizes ovulation timing and reduces sperm–oocyte asynchrony. Integrate with nutritional, environmental, and hormonal management; optimize semen type (conventional vs. sex-sorted).	[202–208]

The administration of GnRH is the primary strategy for stimulating LH release from the pituitary gland [20]. In cows with a dominant follicle that is already in the preovulatory phase but has failed to rupture, GnRH injection can trigger an artificial LH surge and induce ovulation within 24–30 h [167]. The efficacy of GnRH is strongly influenced by the degree of follicle maturation; follicles that have not reached optimal size or physiological competence tend to have a lower response, limiting the effectiveness of GnRH in some individuals [168]. Therefore, the timing of GnRH administration is crucial in the management of delayed ovulation [169]. Reported ovulation or conception success rates following appropriately timed GnRH treatment range from approximately 50–70%, but these rates are context-dependent and can vary with parity, energy balance, postpartum interval, and herd management practices.

Despite its widespread use, GnRH administration has limitations. Repeated or poorly timed injections may result in suboptimal follicular responses, unnecessary treatment costs, and reduced overall protocol efficiency [20]. GnRH is generally preferred when a mature dominant follicle is present and pituitary responsiveness is intact, and is less effective in cows with severe endocrine disruption, systemic disease, or poor metabolic status [169].

Recent studies in North Romania demonstrated that strategic GnRH administration at the time of artificial insemination in cows raised under extensive systems can improve conception rates while being cost-effective, reducing economic losses associated with repeat inseminations [170].

Other therapeutic strategies, such as prostaglandin-based synchronization protocols or combined GnRH-prostaglandin treatments, have also been explored to enhance fertility outcomes in similar extensive farming systems, with variable success and economic implications depending on herd management, drug costs, and labor availability [167].

Human chorionic gonadotropin (hCG) is used as an alternative or complement to GnRH because it acts as an LH agonist with high affinity for LH receptors in the ovaries [170]. Administration of hCG directly stimulates granulosa cell luteinization and facilitates ovulation, with a longer-lasting effect than GnRH [171]. However, repeated hCG use carries specific risks, including antibody formation against hCG, potential ovarian overstimulation, and abnormal luteal development, which limit its routine application. Therefore, hCG is often reserved for cows that fail to respond adequately to GnRH or exhibit clear signs of pituitary insufficiency [164].

Synchronization protocols such as Ovsynch, G6G, and Double Ovsynch offer a more structured therapy strategy [172]. Ovsynch begins with GnRH administration to regulate follicle growth, followed by prostaglandin F2α to lyse the CL, and a second GnRH to trigger coordinated ovulation [173]. Meanwhile, the G6G and Double Ovsynch protocols add a pre-synchronization phase to optimize hormonal responses and standardize follicle status prior to insemination [174]. These protocols generally achieve conception rates of 35–55% per insemination, but success varies with herd health, metabolic status, and management efficiency [175].

## NUTRITIONAL MANAGEMENT

Nutritional management plays a crucial role in preventing and managing delayed ovulation in cattle, particularly during the transition period and early lactation when the animal’s metabolic state is most vulnerable [176]. Inadequate nutrition directly affects ovarian function, follicle growth, and reproductive hormone secretion [177]. Therefore, controlling BCS and meeting micromineral requirements are key strategies to support timely ovulation [47].

Maintaining an optimal BCS is an important indicator of energy balance and metabolic reserves in cows [178]. Cows with a low BCS (≤2.5) in early lactation are at higher risk of prolonged NEB, which decreases IGF-1 and insulin production and reduces ovarian sensitivity to LH, delaying dominant follicle maturation and increasing the risk of persistent follicle formation [179, 180]. Conversely, a high BCS (≥4.0) may cause excessive lipomobilization, fatty liver, and metabolic disorders that disrupt endocrine function [181]. Therefore, targeting a BCS of 3.0–3.5 during the dry period and early lactation is recommended to optimize ovulation and reproductive success [182].

Practical feeding strategies include formulating transition diets that provide controlled energy intake: net energy for lactation of 1.30–1.34 Mcal/kg dry matter during the close-up period, increasing to 1.55–1.65 Mcal/kg dry matter in early lactation, with balanced crude protein levels (14–16%) and an appropriate forage-to-concentrate ratio [183]. Gradual ration adaptation and adequate fiber content help stabilize rumen function and minimize metabolic stress, supporting endocrine recovery postpartum [184].

In addition to energy management, micromineral supplementation is critical, particularly selenium, zinc, copper, manganese, and vitamin E, which support steroidogenesis, antioxidant defense, and follicular development [185]. Organic or chelated mineral sources are preferred due to higher bioavailability and reduced antagonistic interactions. Ensuring adequate mineral status improves ovarian responsiveness to gonadotropins and contributes to more consistent ovulatory outcomes, especially under transition period stress [186].

## ENVIRONMENTAL AND STRESS MANAGEMENT

Environmental management and stress management play a crucial role in supporting cattle reproduction, particularly in preventing delayed ovulation [187]. Environmental stressors, including high temperature, humidity, poor ventilation, and overcrowding, can induce heat stress, which disrupts hormonal balance and ovarian function [188, 189]. Heat stress stimulates cortisol secretion via the HPA axis, suppressing GnRH pulsatility and reducing LH surge amplitude, thereby delaying follicular maturation and increasing the risk of persistent follicles [190]. Additionally, heat stress decreases granulosa cell steroidogenic enzyme activity, resulting in lower estradiol production and less pronounced estrus behavior [191].

Practical animal-based indicators of environmental stress include increased respiration rate, elevated rectal temperature, reduced lying time, altered locomotion, and excessive BCS loss during early lactation. These measures correlate with impaired follicular development, delayed or absent LH surges, and higher incidence of delayed ovulation, providing actionable field-level parameters for reproductive risk monitoring [192].

Effective environmental and welfare management strategies include optimizing housing conditions, ensuring adequate ventilation, providing cooling systems (fans, sprinklers, or integrated shade + forced ventilation + evaporative cooling), and managing stocking density [193]. Among these, integrated cooling systems offer the most effective heat abatement, followed by forced ventilation alone, while shade-only or natural airflow systems are less protective under high heat load conditions. Additional measures such as proper lighting, cleanliness, and comfortable lying areas support physiological and behavioral balance, helping maintain GnRH–LH axis activity and timely ovulation [194, 195].

## POSTPARTUM MANAGEMENT

Postpartum management is a crucial stage in the cow’s reproductive cycle, significantly influencing the timing of ovulation and subsequent pregnancy outcomes [196]. Critical physiological events, including uterine involution and metabolic adaptation, occur predominantly within the first 3–6 weeks postpartum, representing a window where timely interventions are most effective [197]. Implementing a uterine health program and transitional cow management during this period is essential to prevent delayed ovulation [175]. Delayed ovulation is generally considered physiological within the early postpartum period (up to ~20–30 days in milk), but it becomes pathological if the first ovulation fails to occur beyond approximately 40–60 days in milk, depending on production system, metabolic status, and uterine health.

Uterine health programs are designed to accelerate involution and prevent subclinical and clinical infections that disrupt ovarian function [36]. Postpartum infections, such as endometritis or metritis, increase secretion of proinflammatory cytokines (IL-1β, IL-6, and TNF-α) and prostaglandins, which suppress GnRH and LH release, delaying ovulation [150]. Routine monitoring through palpation, ultrasonography, and cytology or neutrophil scoring allows early detection within the critical postpartum window [198]. Integrating uterine health scoring (e.g., vaginal discharge score, ultrasonographic uterine involution, endometrial PMN%) with ovarian monitoring (follicle size, persistence, and CL formation) enables differentiation between delayed ovulation secondary to uterine pathology versus primary endocrine–metabolic causes. Interventions, including selective antibiotic therapy or prostaglandin administration for regression of pathological corpora lutea, help restore uterine health and support timely ovulation [199].

Management of transitioning cows involves careful nutritional, metabolic, and environmental strategies during the three weeks prepartum and three weeks postpartum, when cows are most susceptible to NEB [200]. NEB during this critical window reduces IGF-1, insulin, and glucose levels, impairing follicular maturation and ovarian responsiveness to LH [201]. Management strategies include providing a balanced diet with adequate energy and protein, supplementing essential micronutrients such as selenium, zinc, and vitamin E, and monitoring BCS to prevent excessive fat mobilization [183]. Optimizing housing, ventilation, and heat abatement during this period further supports endocrine recovery and ovulatory competence [42].

By aligning postpartum timelines, uterine health scores, and ovulation monitoring within this critical early lactation window, practitioners can distinguish between physiological and pathological delayed ovulation, allowing timely interventions that improve reproductive efficiency [3]. Systematic uterine health programs and optimized transition cow management accelerate first postpartum ovulation, reduce the incidence of delayed ovulation, and increase the likelihood of successful conception, thereby enhancing herd level productivity [11].

## ASSISTED REPRODUCTION TECHNOLOGIES

Assisted reproduction technologies are a crucial strategy for addressing delayed ovulation in cattle, particularly in increasing the effectiveness of AI and pregnancy success rates [202]. It is important to note that these technologies are intended to complement, rather than replace, optimal nutritional, environmental, and hormonal management practices. Accurate timing of insemination is key, as delayed ovulation can lead to a lack of synchrony between mature oocytes and fertilizing sperm [203]. Understanding follicular dynamics, estrus behavior, and the hormonal profile of each individual cow allows for optimal insemination timing, thus increasing the chance of fertilization [204].

In cows affected by delayed ovulation, conception rates following spontaneous AI-based solely on visual estrus detection are generally lower compared to hormonally synchronized programs, due to mistimed insemination relative to the delayed LH surge and ovulation [202]. In contrast, fixed-time artificial insemination (FTAI) protocols have been shown to improve conception rates in these cows by standardizing ovulation timing and reducing the risk of sperm–oocyte asynchrony. Several studies report higher or more consistent pregnancy rates with FTAI compared to spontaneous AI in herds with a high prevalence of ovulatory disorders, particularly under conditions of poor estrus expression or intensive management systems [204].

FTAI is a practical application of assisted reproductive technology to address individual differences in ovulation timing [205]. This method utilizes hormonal protocols, such as Ovsynch or Double Ovsynch, to synchronize oocyte release with insemination at a predetermined time without waiting for natural signs of estrus [206]. FTAI is most effective when integrated with ongoing herd level management, including optimal nutrition, metabolic monitoring, and heat stress mitigation. This approach is particularly useful in cows with delayed ovulation because it reduces reliance on manual estrus detection, which is prone to error and behavioral variability [6]. Through FTAI, ovulation can be triggered with a combination of GnRH and prostaglandins, resulting in simultaneous ovulation across the herd, improving overall reproductive efficiency [207].

The choice of semen type is also an important consideration when applying ART in cows with delayed ovulation. Conventional semen generally yields higher conception rates than sex-sorted semen due to its higher sperm concentration and better resilience to suboptimal timing [206]. In delayed ovulation cases, the narrower fertilization window and reduced sperm longevity associated with sex-sorted semen may further compromise pregnancy outcomes if insemination timing is not optimal. Thus, proper semen selection should be considered alongside ART protocols to maximize reproductive outcomes in herds with variable ovulatory responses [202].

Combining assisted reproductive technology with optimized nutritional, environmental, and hormonal management, along with follicular monitoring and hormonal profiling, provides a comprehensive strategy for managing delayed ovulation [208]. Such an integrated approach allows practitioners to select the most appropriate insemination strategy (spontaneous AI versus FTAI) and semen type based on ovulatory status, herd management level, and production goals, thereby improving insemination success and overall reproductive performance in both dairy and beef cattle systems [164].

## FUTURE DIRECTIONS AND RESEARCH GAPS

Although knowledge about delayed ovulation in cattle has made significant progress, several aspects still require further research to improve reproductive efficiency in the modern livestock industry [1]. To sharpen research focus, future studies should prioritize 2–3 key questions: (i) what physiological or management thresholds distinguish adaptive postpartum ovulatory delay from pathological delayed ovulation; (ii) which combinations of metabolic, inflammatory, and endocrine biomarkers provide the highest predictive accuracy for identifying cows at risk; and (iii) how the timing of interventions, nutritional, hormonal, or environmental, can be optimized to minimize unnecessary treatment while maximizing fertility outcomes.

One promising area of research is the development of predictive biomarkers [209]. Molecular and metabolic biomarkers, including inflammatory mediators, reproductive hormones, and energy metabolites such as NEFA and BHB, have the potential to predict the risk of delayed ovulation before clinical symptoms appear [83]. Future work should focus on validating standardized biomarker panels, establishing practical cutoff values, and assessing their predictive performance under different production systems, parity, physiological stages, and genetic backgrounds. By identifying a panel of sensitive and specific biomarkers, early detection of ovulatory disorders becomes possible, allowing for more timely management and hormonal therapy interventions [210].

Another key avenue is the integration of artificial intelligence (AI) and precision livestock farming (PLF) technologies. AI systems can analyze real-time data on cow behavior, locomotion, body temperature, and feeding patterns to predict estrus and ovulation with greater accuracy than conventional observation-based methods [211, 212]. PLF combines sensors, wearable devices, and AI-based data analysis to provide individualized monitoring of reproductive status [213]. This approach enables tailoring nutritional, hormonal, and environmental management to each cow, reducing estrus cycle variability and the incidence of delayed ovulation.

However, practical implementation challenges remain. High initial costs, infrastructure requirements, and data management complexity limit adoption in low-resource or smallholder farms. Future research should therefore emphasize simplified decision-support tools, low-cost sensor technologies, and management-based indicators that can be applied in extensive or resource-limited production systems [214].

Finally, welfare and ethical considerations must be integrated into future strategies. Excessive or routine hormonal manipulation may increase stress, disrupt endocrine balance, and raise societal concerns regarding sustainable livestock production. Research should evaluate long-term welfare outcomes and prioritize preventive strategies, such as optimized nutrition, environmental management, and early disease detection, before resorting to hormonal interventions [215].

While promising, further studies are needed to refine predictive algorithms, validate biomarkers across diverse management and genetic contexts, evaluate cost–benefit and welfare implications, and integrate these tools effectively into commercial-scale livestock systems [216].

## LIMITATIONS

Despite providing a comprehensive overview of delayed ovulation in cattle, several limitations of this review should be acknowledged. First, the literature on this topic spans more than two decades (2000–2025), and while seminal and foundational studies are included, some earlier findings may not fully reflect the most recent management practices or technological advances. Second, most available studies are descriptive or observational, and controlled intervention trials are limited, which constrains the ability to draw strong causal inferences regarding risk factors or management effectiveness. Third, there is heterogeneity in hormonal thresholds, diagnostic criteria, and measurement techniques across studies, making direct comparisons challenging and complicating the development of universally applicable recommendations. Fourth, the review is narrative rather than systematic, which may introduce selection bias despite structured literature searches and predefined inclusion criteria. Finally, translational challenges exist in applying findings from controlled research settings to diverse farm conditions, production systems, and breeds, where environmental, nutritional, and management variables can influence outcomes differently.

These limitations highlight the need for standardized protocols, multi-center studies, and context-specific evaluation of reproductive interventions. Addressing these gaps in future research will strengthen the evidence base and provide more robust guidance for veterinarians and livestock practitioners in optimizing reproductive management and improving fertility efficiency in cattle.

## CONCLUSION

Delayed ovulation in cattle represents a clinically important reproductive disorder characterized by postponed oocyte release following estrus, primarily driven by disruptions in the HPO axis. This review synthesizes current evidence on its physiological basis, multifactorial etiology (nutritional, environmental, animal-related, endocrine–metabolic, and infectious-inflammatory), clinical manifestations including follicular persistence and weak estrus expression, diagnostic approaches (ultrasonography, hormonal profiling, behavioral monitoring, and emerging biomarkers), and integrated management strategies encompassing hormonal therapies, nutritional optimization, environmental control, postpartum care, and ART. Key impacts include reduced conception rates, prolonged calving intervals, increased repeat breeding, and substantial economic losses estimated at USD 50–150 per affected cow per cycle, with greater effects observed in high-producing dairy systems.

The findings underscore the importance of early identification and targeted interventions. Routine integration of ovarian ultrasonography with metabolic monitoring (e.g., NEFA, BHB, IGF-1) and body condition scoring during the transition and early postpartum periods enables timely differentiation between physiological and pathological delays. Strategic use of GnRH-based synchronization protocols, FTAI, optimized transition diets targeting BCS 3.0–3.5, and heat stress abatement can significantly improve ovulation timing and herd fertility. Herd level implementation of these evidence-based practices supports more sustainable reproductive management across dairy and beef production systems.

This integrative review provides a comprehensive, cross-disciplinary synthesis that clearly distinguishes delayed ovulation from related conditions such as COD, silent heat, and anovulation. It bridges physiological mechanisms with practical field applications and highlights system-specific considerations, offering actionable guidance for veterinarians and livestock practitioners.

As a narrative review spanning 2000–2025 literature, it is subject to the inherent heterogeneity of study designs, diagnostic criteria, and production contexts. The predominance of observational data limits strong causal conclusions, and translational applicability may vary across diverse farming systems and breeds.

Priority research should focus on validating predictive biomarker panels, developing cost-effective PLF tools adaptable to resource-limited settings, establishing clear thresholds separating adaptive from pathological delays, and evaluating long-term welfare and economic outcomes of preventive versus therapeutic strategies. Multi-center, controlled trials and standardized protocols will be essential to strengthen the evidence base.

In conclusion, addressing delayed ovulation through integrated, evidence-based approaches holds substantial potential to enhance reproductive efficiency, reduce economic losses, and promote sustainable cattle production. By translating current knowledge into practical on-farm strategies while advancing targeted research, veterinarians and producers can achieve more precise, welfare-conscious, and productive reproductive management in modern livestock systems.

## DATA AVAILABILITY

The supplementary data can be made available from the corresponding author upon request.

## GENERATIVE AI DECLARATION

The authors declare that the figures are conceptual illustrations generated using AI tools for visualization purposes only. No experimental data are presented. They also declare that AI was used to edit the paper's grammar. The authors have reviewed and verified the figures and grammar editing for scientific accuracy.

## AUTHORS’ CONTRIBUTIONS

LP, ARK, AB, and IM: Drafted the manuscript. BPP, RZA, and WW: Revised and edited the manuscript. SR, RR, DFK, and GMOPC: Contributed to manuscript preparation and critically reviewed the manuscript. AOA, ODP, and JJ: Revised and edited the references. All authors have read and approved the final version of the manuscript.
